# Limited clinical relevance of systemic inflammatory indices in idiopathic epiretinal membrane: a retrospective case–control study

**DOI:** 10.1186/s12886-026-04842-5

**Published:** 2026-04-24

**Authors:** Enes Toklu, Gözde Göknar, Seda Karaca Adıyeke

**Affiliations:** Department of Ophthalmology, Faculty of Medicine, University of Bakırçay, İzmir Çiğli Training and Research Hospital, İzmir, Turkey

**Keywords:** Idiopathic epiretinal membrane, Neutrophil-to-lymphocyte ratio, Platelet-to-lymphocyte ratio, Systemic immune-inflammation index, Vitreoretinal interface

## Abstract

**Background:**

Idiopathic epiretinal membrane (iERM) is a common age-related vitreoretinal disorder characterized by fibrocellular proliferation on the inner limiting membrane. Although systemic inflammation has been suggested to play a potential role, the diagnostic value of systemic inflammatory indices such as neutrophil-to-lymphocyte ratio (NLR), platelet-to-lymphocyte ratio (PLR), and systemic immune-inflammation index (SII) remains unclear. This study aimed to evaluate whether the relationships of iERM with clinical and hematological parameters, including age, gender, systemic inflammatory indices and common comorbidities, provide clinically significant additional information.

**Methods:**

This retrospective case–control study included 59 patients with iERM and 56 healthy control subjects. The NLR, PLR, SII were compared between groups. Multivariable logistic regression analyses were performed to identify independent associations with iERM, and receiver operating characteristic (ROC) analyses were used to assess the discriminative performance of systemic inflammatory indices. An additional age-matched sensitivity analysis was conducted to address potential age-related confounding.

**Results:**

Patients with iERM were significantly older than controls (*p* < 0.001). NLR, PLR, SII, neutrophil, lymphocyte, and platelet values showed no significant difference between groups (all *p* > 0.05). These findings remained consistent in the age-matched sensitivity analysis, in which 36 patients with iERM were matched to 36 controls, demonstrating no significant differences in systemic inflammatory indices or hematologic parameters between matched groups (all *p* > 0.05). Comorbidities such as diabetes mellitus and hypertension were not independently associated with iERM (*p* = 0.918). In multivariable logistic regression models, age remained the only independent predictor of iERM (Model-0: AUC = 0.723). The inclusion of NLR, PLR, or SII did not significantly improve model performance (AUC < 0.02, higher AIC/BIC, lower HL-p). ROC analyses demonstrated low discriminatory ability (NLR: 0.567, PLR: 0.522, SII: 0.568).

**Conclusion:**

Confirming the limited clinical significance of these readily available biomarkers will be important to avoid unnecessary clinical use of such markers in iERM and present findings may help guide future research toward local retinal mechanisms rather than systemic inflammatory biomarkers.

## Introduction

Epiretinal membrane (ERM) is a common cause of visual impairment characterized by the presence of an avascular fibrocellular contractile layer on the vitreoretinal interface, with an estimated prevalence of approximately 6–7% in the general population [1]. The condition disrupts the normal arrangement of photoreceptors, leading to visual distortion (metamorphopsia) and decreased visual acuity [2, 3]. Histologically, ERMs consist of an inner cellular component and an outer layer composed of extracellular matrix (ECM) proteins [3]. Increased levels of cytokines such as transforming growth factor (TGF) within the local microenvironment promote myofibroblastic transformation and subsequent membrane contraction [4, 5].

Vitreous and tissue analyses have revealed significantly elevated levels of various cytokinesincluding fibroblast growth factor (FGF), nerve growth factor (NGF), and vascular endothelial growth factor (VEGF) in individuals with idiopathic epiretinal membrane (iERM), suggesting that local inflammatory mechanisms play a critical role in its pathogenesis [6, 7]. In the assessment of systemic inflammation, simple, inexpensive, and reproducible biomarkers derived from complete blood count parameters, such as the neutrophil-to-lymphocyte ratio (NLR) and platelet-to-lymphocyte ratio (PLR), have gained increasing attention [8–10]. The NLR, which reflects both neutrophilia (inflammation) and lymphopenia (physiologic stress), has demonstrated prognostic value in various systemic disorders, including malignancies and cardiovascular diseases [11, 12], as well as in ophthalmic conditions such as age-related macular degeneration and glaucoma [13, 14]. Regarding iERM, some studies have reported significantly higher NLR values in affected patients compared with healthy controls [15]. However, the existing evidence in the literature remains inconsistent, and several studies have failed to confirm this association [15, 16]. Moreover, the role of more comprehensive hematologic indicators such as mean platelet volume (MPV), a marker of platelet activation, and the systemic immune inflammation index (SII), which integrates neutrophil, platelet, and lymphocyte counts has not been thoroughly investigated in iERM [15]. To address these inconsistencies, the present study aimed to evaluated whether the relationships of iERM with clinical and hematological parameters, including age, gender, systemic inflammatory indices (NLR, PLR, and SII), and common comorbidities such as DM and HT, provide clinically significant additional information.

### Study design

This retrospective case–control study was conducted at the Department of Ophthalmology, Bakırçay University Çiğli Training and Research Hospital, between January 2023 and December 2024. The study included patients who were diagnosed with iERM and underwent pars plana vitrectomy with epiretinal membrane and internal limiting membrane (ERM–ILM) peeling. Data were collected retrospectively from patient medical records and the hospital information management system following approval from the Bakırçay University Ethics Committee (approval no: 2024/1860). The study was carried out in accordance with the principles of the Declaration of Helsinki and applicable local ethical regulations.

### Patient selection

The study population was divided into two cohorts. The iERM group included 59 patients who underwent pars plana vitrectomy with combined epiretinal membrane and internal limiting membrane peeling. In this group, complete blood count (CBC) parameters were obtained within one week before surgery as part of the routine preoperative assessment. Patients with clinical evidence of acute infection at the time of preoperative assessment were not considered eligible for surgery. The control group comprised 56 subjects who had presented to the ophthalmology outpatient clinic within the preceding six months and had undergone CBC testing during the same visit and showed no macular pathology on spectral-domain optical coherence tomography or evidence of systemic inflammatory disease. The diagnosis of iERM was established clinically by slit-lamp biomicroscopic fundus examination and confirmed by SD-OCT (Spectralis; Heidelberg Engineering, Heidelberg, Germany). To exclude vascular and inflammatory ocular conditions, including diabetic retinopathy, retinal vein occlusion (RVO), uveitis, and ocular malignancy, all patients underwent fundus fluorescein angiography (FFA). Although CBC values were obtained within 6 months, individuals with known inflammatory diseases or acute infections were excluded to minimize potential bias. Exclusion criteria were a history of intraocular surgery, ocular trauma, retinal detachment, uveitis, diabetic retinopathy, retinal vein occlusion, age-related macular degeneration, high myopia (> 6 diopters), autoimmune or cardiovascular disease, malignancy, and the use of chemotherapeutic agents, iron preparations, or corticosteroids. In addition, subjects with any anterior or posterior segment pathology, such as synechiae or intraocular inflammatory reaction, or with glaucoma were excluded from both groups. Other potential confounders such as lens status, previous cataract surgery, and PVD were not available for all patients.

### Data collection

Hematologic data were retrospectively obtained from the hospital information system. Venous blood samples were collected from the antecubital vein and analyzed within 30 min using an automated hematology analyzer (Mindray BC-2800, Shenzhen, China). Blood samples from the patient group were obtained as part of their routine preoperative evaluation. Recorded parameters included neutrophil, lymphocyte, and platelet counts (×10³/µL). These variables were used to calculate the following systemic inflammatory indices: neutrophil-to-lymphocyte ratio (NLR = neutrophil count / lymphocyte count), platelet-to-lymphocyte ratio (PLR = platelet count / lymphocyte count), and systemic immune-inflammation index (SII = [neutrophil count × platelet count] / lymphocyte count).

### Statistical analysis

The distribution of continuous variables was assessed using the Shapiro–Wilk and D’Agostino K² tests for normality. Non-normally distributed variables, including NLR, PLR, SII, and neutrophil and platelet counts, were compared using the Mann–Whitney U test, whereas normally distributed variables (lymphocyte) were analyzed using the independent samples t-test. Descriptive statistics were expressed as mean ± standard deviation (SD) or median [interquartile range, IQR], as appropriate. Categorical variables were analyzed using the Chi-square test or Fisher’s exact test. Associations between comorbid conditions and the presence of iERM were expressed as odds ratios (ORs) with 95%confidence intervals (CIs). Multivariable logistic regression models incorporating age, sex, and hematologic inflammatory indices were constructed to identify factors independently associated with iERM. Model calibration was assessed using the Hosmer–Lemeshow (HL) goodness-of-fit test, and model performance was compared using the Akaike (AIC) and Bayesian (BIC) information criteria. Diagnostic performance was evaluated using receiver operating characteristic (ROC) curve analysis. The area under the curve (AUC), 95% CI, optimal cutoff point (Youden index), sensitivity, specificity, positive predictive value (PPV), and negative predictive value (NPV) were reported. To minimize the potential confounding effect of age, an age-matched sensitivity analysis was performed. Patients with idiopathic epiretinal membrane were matched 1:1 with control subjects using a nearest-neighbor approach within a ± 3-year caliper without replacement. Only matched pairs were included in this analysis, resulting in 36 matched pairs. Statistical significance was defined as a p-value < 0.05.

## Results

A total of 115 individuals were included, comprising 59 patients with iERM and 56 control subjects. Patients with iERM were significantly older than controls (68.51 ± 4.89 vs. 61.96 ± 11.31 years, *p* < 0.001). Sex distribution and the prevalence of systemic comorbidities, including diabetes mellitus (DM) and hypertension (HT), were comparable between groups (*p* = 0.780 and *p* = 0.918, respectively), indicating balanced baseline characteristics (Table 1).

**Table 1:** Baseline characteristics of participants

Variable	Without iERM (*n*, %)	With iERM (*n*, %)	Total (*n*)	*p*-value
**Sex**				0.780
Male	24 (42.9)	24 (40.7)	48	
Female	32 (57.1)	35 (59.3)	67	
**Comorbidities**				0.918
None	40 (71.4)	44 (74.6)	84	
Diabetes mellitus (DM)	9 (16.1)	8 (13.6)	17	
Hypertension (HT)	7 (12.5)	7 (11.9)	14	

Data are presented as number (percentage). Comparisons were made using the Chi-square test. iERM: idiopathic epiretinal membrane; DM: diabetes mellitus; HT: hypertension

In the association analysis, neither DM (OR = 0.82, 95% CI: 0.29–2.30, *p* = 0.795) nor HT (OR = 0.94, 95% CI: 0.31–2.88, *p* = 1.000) was significantly associated with iERM. Overall comorbidity distribution was also similar between groups (*p* = 0.918) (Table 2).

**Table 2:** Distribution of comorbidities according to the presence of iERM

Comorbidity	Without İERM (*n*, %)	With iERM (*n*, %)	Odds Ratio (OR)	95% Confidence Interval (CI)	Fisher’s *p*-value
Diabetes mellitus (DM)	9 (16.1%)	8 (13.6%)	0.82	0.29–2.30	0.795
Hypertension (HT)	7 (12.5%)	7 (11.9%)	0.94	0.31–2.88	1.000
None	40 (71.4%)	44 (74.6%)	1.17	0.51–2.68	0.834
Overall comparison	56 (100%)	59 (100%)	—	—	0.918

Data are presented as number (percentage). Fisher’s exact test was used for categorical comparisons. OR: odds ratio; CI: confidence interval; DM: diabetes mellitus; HT: hypertension; iERM: idiopathic epiretinal membrane

Comparison of continuous variables demonstrated that, aside from age, no significant differences were observed between groups in systemic inflammatory indices (NLR, PLR, and SII) or individual hematologic parameters (all *p* > 0.05) (Table 3).

**Table 3:** Comparison of continuous variables according to the presence of iERM

Variable	Without iERM (*n* = 56)Mean ± SD	With iERM (*n* = 59)Mean ± SD	U / t	Z	*p*-value
Age (years)	61.96 ± 11.31	68.51 ± 4.89	949.0	−3.94	< 0.001*
NLR	1.88 ± 0.68	2.03 ± 0.66	1432.0	−1.23	0.218
PLR	123.38 ± 46.90	130.17 ± 50.61	1580.0	−0.40	0.687
SII	486.72 ± 236.57	534.40 ± 233.09	1428.0	−1.25	0.210
Neutrophil (10³/µL)	3.97 ± 1.07	4.14 ± 1.19	1573.5	−0.44	0.660
Platelet (10³/µL)	254.86 ± 63.15	257.54 ± 67.05	1631.5	−0.12	0.909
Lymphocyte (10³/µL)	2.25 ± 0.64	2.15 ± 0.63	−0.83	—	0.410**

*Values are expressed as mean ± standard deviation (SD). Continuous variables were analyzed using the Mann–Whitney U test unless otherwise specified.**Independent samples t-test; *p* < 0.05 was considered statistically significant

NLR: neutrophil-to-lymphocyte ratio; PLR: platelet-to-lymphocyte ratio; SII: systemic immune-inflammation index

In multivariable logistic regression analyses, age remained the only independent predictor of iERM. The inclusion of systemic inflammatory indices (NLR, PLR, and SII) did not improve model performance, as indicated by minimal changes in AUC, higher AIC and BIC values, and no improvement in calibration. These findings further support that systemic inflammatory indices have limited clinical utility in the prediction of iERM (Table 4).

**Table 4:** Comparison of logistic regression model performance for predicting iERM

Model	*n*	AUC	AIC	BIC	HL-*p*
**Model-0**: Age + Sex	115	0.723	156.115	164.350	0.046
**Model-1**: Age + Sex + NLR	115	0.725	158.059	169.038	0.023
**Model-2**: Age + Sex + PLR	115	0.713	158.157	169.136	0.007
**Model-3**: Age + Sex + NLR + PLR + SII	115	0.713	161.743	178.213	0.018

AUC: area under the receiver operating characteristic curve; AIC: Akaike information criterion; BIC: Bayesian information criterion; HL-p: Hosmer–Lemeshow goodness-of-fit test p-value; NLR: neutrophil-to-lymphocyte ratio; PLR: platelet-to-lymphocyte ratio; SII: systemic immune-inflammation index. A *p* < 0.05 was considered statistically significant

Consistent with these findings, ROC analysis showed that all inflammatory indices had poor discriminative ability, with AUC values close to 0.5, indicating performance comparable to random classification. Sensitivity, specificity, positive predictive value (PPV), and negative predictive value (NPV) were all low to moderate, indicating limited diagnostic performance. These findings indicate that systemic inflammatory indices are not suitable as diagnostic biomarkers for iERM supporting the limited diagnostic utility of these markers (Table 5).

**Table 5:** Receiver operating characteristic (ROC) analysis results for hematologic inflammatory biomarkers

Biomarker	AUC (95% CI)	Optimal Cutoff (Youden Index)	Sensitivity	Specificity	PPV	NPV
NLR	0.567 (0.461–0.668)	2.07	0.475	0.696	0.622	0.557
PLR	0.522 (0.409–0.631)	151.53	0.356	0.804	0.656	0.542
SII	0.568 (0.459–0.675)	468.25	0.559	0.571	0.579	0.552

Data are presented as values derived from ROC curve analysis. AUC: area under the receiver operating characteristic curve; CI: confidence interval; PPV: positive predictive value; NPV: negative predictive value; NLR: neutrophil-to-lymphocyte ratio; PLR: platelet-to-lymphocyte ratio; SII: systemic immune-inflammation index

In the age-matched sensitivity analysis, 36 patients with iERM were matched with 36 controls. No significant differences were observed in systemic inflammatory indices or hematologic parameters (all *p* > 0.05), confirming that the lack of association was independent of age (Table 6).

**Table 6:** Age-matched comparison of systemic inflammatory indices between iERM patients and controls

Variable	Controls (*n* = 36)	iERM (*n* = 36)	*p*-value
Age (years)	Matched	Matched	—
NLR	Median (IQR)	Median (IQR)	0.133
PLR	Median (IQR)	Median (IQR)	0.427
SII	Median (IQR)	Median (IQR)	0.148
Neutrophil (×10³/µL)	Median (IQR)	Median (IQR)	0.978
Lymphocyte (×10³/µL)	Median (IQR)	Median (IQR)	0.267
Platelet (×10³/µL)	Median (IQR)	Median (IQR)	0.919

Data are presented as median (interquartile range). Age matching was performed using a 1:1 nearest-neighbor approach within a ± 3-year caliper. Comparisons between groups were conducted using the Mann–Whitney U test. NLR: neutrophil-to-lymphocyte ratio; PLR: platelet-to-lymphocyte ratio; SII: systemic immune-inflammation index; iERM: idiopathic epiretinal membrane

**Fig. 1:**
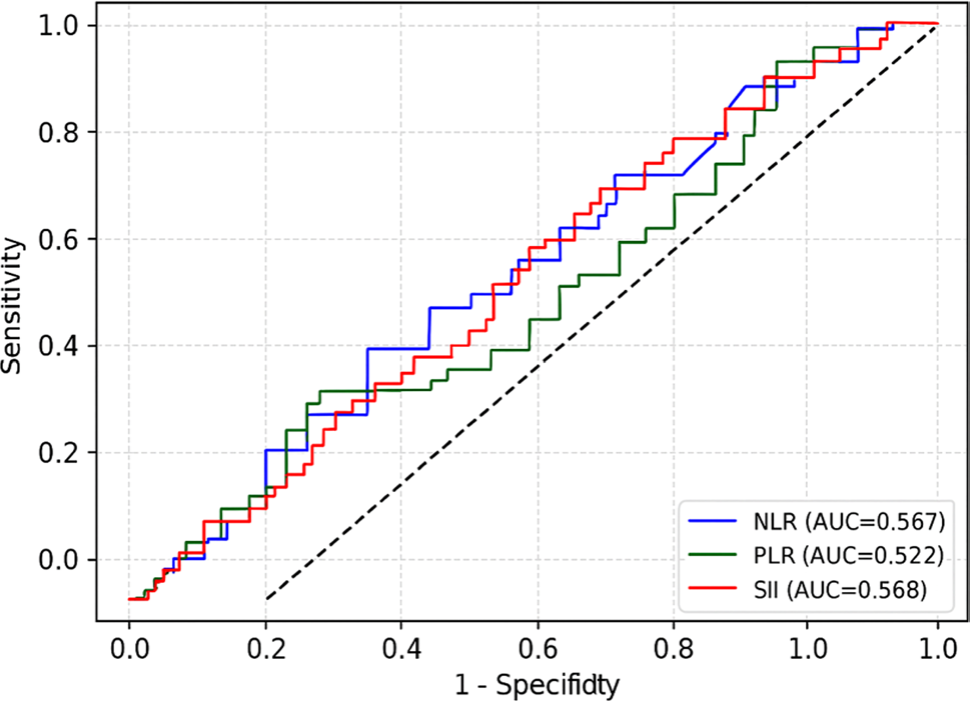
ROC Curves: NLR, PLR, SII

ROC curve analysis demonstrated poor discriminative performance of all systemic inflammatory indices, with AUC values close to 0.5 and curves approximating the diagonal reference line, indicating performance comparable to random classification. Accordingly, NLR, PLR, and SII showed no clinically meaningful diagnostic utility in iERM (Fig. 1).

**Fig. 2:**
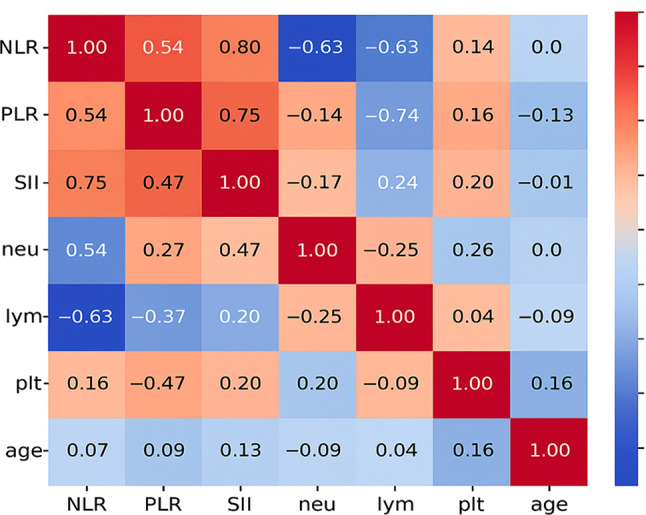
Spearman Correlation Heatmap

Spearman correlation analysis revealed expected interrelationships among inflammatory indices and their hematologic components. While NLR was positively correlated with neutrophil count and negatively correlated with lymphocyte count, no significant correlation was observed between age and any inflammatory index, suggesting independence from age-related effects (Fig. 2).

**Fig. 3:**
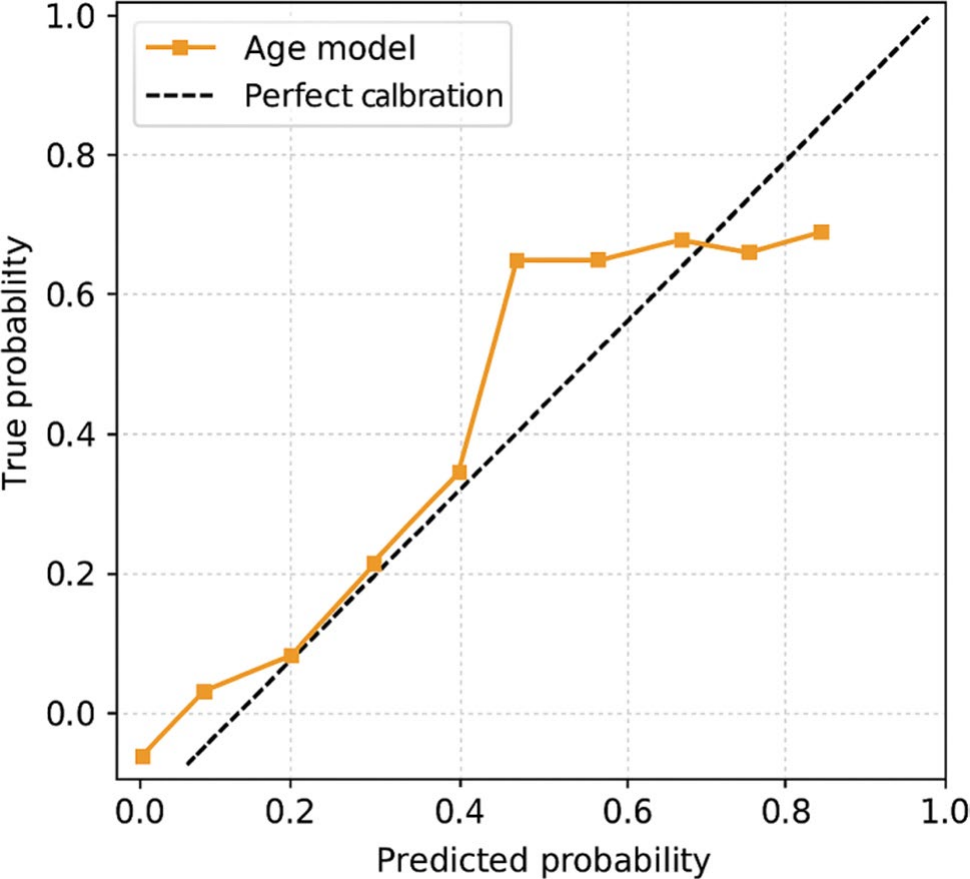
Calibration Curve (Age Model)

Calibration analysis showed that the age-based model achieved acceptable agreement between predicted and observed probabilities, with minor deviations at higher probability ranges, indicating reasonable but imperfect calibration (Fig. 3).

Taken together, our results consistently indicate that systemic inflammatory indices, including NLR, PLR, and SII, do not contribute to the identification or prediction of iERM beyond age. The concordant findings across multiple analytical approaches including group comparisons, multivariable modeling, ROC analysis, and age-matched sensitivity analysis strengthen the robustness of this conclusion and suggest that systemic inflammation plays a limited role in the pathogenesis of iERM.

## Discussion

This study evaluated whether the relationships of iERM with clinical and hematological parameters, including age, gender, systemic inflammatory indices (NLR, PLR, and SII), and common comorbidities such as DM and HT, provide clinically significant additional information.

Patients with iERM were significantly older than controls, consistent with previous studies identifying age as a major risk factor [10, 12, 17, 18]. Aging-related changes such as vitreous degeneration, posterior vitreous detachment, and increased glial proliferation may contribute to iERM development [19, 20]. No significant difference in sex distribution was observed between groups. In line with our findings, several studies have reported no meaningful association between sex and iERM [1], although some have suggested a possible female predominance [20, 21]. These inconsistencies may be related to differences in study populations and methodological factors.

Inflammation-related biomarkers derived from routine complete blood count parameters (such as NLR, PLR, and SII) have recently gained attention in ophthalmology because they are inexpensive, widely available, and have been reported to be associated with several ocular diseases. Our study contributes to the literature by performing a comprehensive evaluation using multivariable regression modeling, ROC curve analysis, and an age-matched sensitivity analysis to address the important confounding effect of age. Across all analyses, systemic inflammatory indices showed minimal discriminative ability and did not improve predictive models beyond age alone. This is supported by multivariable analyses showing age as the only independent factor, as well as by the low AUC values observed for NLR, PLR, and SII. Previous studies evaluating inflammatory blood indices in iERM have reported conflicting results [9, 10, 12, 13]. Dikkaya et al. [15]. reported significantly higher NLR values in patients with iERM, while Uzlu et al. [16] also found elevated NLR values, although PLR was not significantly different between groups. Similarly, Demir et al. [22] reported higher NLR, PLR, and MPV levels in iERM patients compared with controls. Qin et al. [23] further reported significantly elevated inflammatory indices, including MLR, NLR, and PLR, with moderate diagnostic performance. In contrast, the null findings in our study remained consistent not only in the primary analyses but also in the age-matched sensitivity analysis, suggesting that previously reported associations may be influenced by residual confounding, particularly age, as well as differences in patient selection, disease severity, sample size, and preanalytical variability.

Systemic diseases such as DM and HT are known to be associated with chronic inflammation and may affect retinal microcirculation through endothelial dysfunction [18, 24]. However, their relationship with iERM remains inconsistent in the literature. While some epidemiological studies have suggested that DM may be associated with an increased prevalence of iERM [20], other investigations have not confirmed this association [24]. In our study, neither DM nor HT emerged as an independent factor associated with iERM. Taken together with the dominant effect of age in the multivariable analyses, these findings suggest that age-related vitreoretinal alterations may be more relevant than systemic inflammatory burden related to comorbid disease in this setting.

Age is a well-established determinant of iERM, and inadequate adjustment may lead to spurious associations between inflammatory indices and disease presence [25]. In the present study, multivariable modeling and age-matched analysis consistently showed that systemic inflammatory markers do not provide additional discriminatory value beyond age, suggesting that previously reported associations may reflect residual confounding rather than true pathophysiological links.

Given that iERM is primarily driven by localized vitreoretinal processes, peripheral blood-based inflammatory indices may not adequately reflect the relevant intraocular microenvironment [26–28]. As emphasized by Sertoglu et al., [9] the clinical applicability of NLR may be substantially affected by preanalytical factors, including sampling conditions and physiological fluctuations. Accordingly, our findings indicate that these readily available biomarkers have limited clinical utility in iERM. The main contribution of this study, therefore, lies in providing a more methodologically robust evaluation that clarifies the limited role of systemic inflammatory indices when key confounders, particularly age, are appropriately addressed.

Another notable finding of our study was the limited discriminative ability of NLR, PLR, and SII in ROC curve analysis. The AUC values for these biomarkers were close to 0.5, indicating performance comparable to random classification. Similarly, the inclusion of these indices in multivariable predictive models did not improve model performance beyond age alone. From a clinical perspective, these findings suggest that systemic inflammatory indices derived from routine blood tests are unlikely to contribute substantially to the diagnostic evaluation or risk stratification of patients with idiopathic epiretinal membrane. Similarly, Qin et al. [23] and Demir et al. [22] reported low diagnostic discrimination for these parameters in identifying iERM.

NLR and PLR have been widely studied as inflammatory biomarkers in various ocular and systemic diseases; [9, 12, 16, 23, 25] however, in conditions characterized by localized and low-grade inflammation such as iERM, their sensitivity may be limited. Preanalytical variability and physiological fluctuations may further influence these markers [1, 29–31]. In addition, the absence of differences across comorbidity subgroups suggests that systemic inflammatory burden is not adequately reflected in iERM. These findings support the concept that iERM is primarily a localized vitreoretinal process, rather than a manifestation of systemic inflammation.

Our iERM cohort consisted exclusively of surgically treated patients, likely representing more advanced and symptomatic cases rather than the full disease spectrum. Therefore, the findings may not be fully generalizable to milder, conservatively managed iERM. Notably, even in this clinically significant subgroup, CBC-derived inflammatory indices did not provide meaningful discriminatory value beyond age, further supporting their limited clinical relevance in iERM.

Taken together, our findings suggest that systemic inflammatory indices derived from routine blood tests do not provide clinically meaningful information in iERM. Rather than introducing a novel biomarker, the present study contributes by providing a methodologically robust evaluation that clarifies the limited role of these indices when key confounders, particularly age, are appropriately addressed. Confirming the limited clinical significance of these readily available biomarkers will be important to guide future research and avoid unnecessary clinical use of such markers in iERM.

This study has several limitations. First, its retrospective case-control design precludes direct inference of causal relationships between variables. Second, as a single-center study conducted at a tertiary referral center, the findings may not be fully generalizable to broader iERM populations or to community-based settings. Third, the relatively limited sample size may have reduced statistical power, particularly for subgroup analyses involving comorbid conditions such as DM and HT. In addition, the iERM cohort consisted exclusively of surgically treated patients and therefore likely represented clinically significant, more symptomatic cases rather than the full spectrum of iERM.

Another limitation is that the hematologic parameters evaluated were derived from systemic circulation and may not adequately reflect the localized inflammatory microenvironment of iERM. Moreover, due to the retrospective nature of the study, DM and HT were recorded only as dichotomous variables and were not further stratified according to disease duration, severity, or treatment status, all of which may have subtly influenced CBC-derived inflammatory indices. Likewise, Another limitation of this study is the lack of adjustment for ocular factors such as lens status, prior cataract surgery, and posterior vitreous detachment (PVD), which are known to be associated with iERM and may correlate with age. Due to the retrospective nature of the study, these variables were not consistently available for all participants. Therefore, residual confounding cannot be entirely excluded. Potential confounding factors such as smoking status, body mass index, and lipid profile were also not controlled.

Finally, CBC-derived inflammatory indices are influenced by several preanalytical and physiological factors, including circadian variation, hydration status, and subclinical intercurrent illness. Although complete blood count measurements in the iERM group were obtained within one week prior to surgery as part of routine preoperative evaluation, and patients with clinical evidence of acute infection were not considered eligible for surgery, residual variability related to these factors cannot be excluded. Such variability may have further reduced the ability to detect subtle between-group differences. Therefore, larger multicenter prospective studies incorporating intraocular biomarkers and more comprehensive systemic and ocular characterization are needed to validate and extend the present findings.

Conclusion, previous studies have reported mixed or negative findings regarding the association between systemic inflammatory indices and iERM. However, many of these studies did not adequately account for confounding factors, particularly age, which is a well-established determinant of iERM.In this context, the present study contributes to the literature by providing a more methodologically robust evaluation through multivariable regression and age-matched sensitivity analysis. The consistent findings across these approaches demonstrate that the lack of association between inflammatory indices and iERM is independent of age. Therefore, rather than merely replicating previous findings, our study helps clarify inconsistencies in the literature and reinforces the limited clinical utility of systemic inflammatory markers in iERM.

## Data Availability

The datasets generated and/or analyzed during the current study are available from the corresponding author on reasonable request.

## Abbreviations

iERM: Idiopathic epiretinal membrane
NLR: Neutrophil-to-lymphocyte ratio
PLR: Platelet-to-lymphocyte ratio
SII: Systemic immune-inflammation index
ROC: Receiver operating characteristic
AUC: Area under the curve
AIC: Akaike information criterion
BIC: Bayesian information criterion
HL: Hosmer–Lemeshow
OR: Odds ratio
CI: Confidence interval
PPV: Positive predictive value
NPV: Negative predictive value
SD: Standard deviation
IQR: Interquartile range
DM: Diabetes mellitus
HT: Hypertension
